# Supplementary figures and images for: Cost-effectiveness of screening for chronic kidney disease using a cumulative eGFR-based statistic
Source: PLoS One. 2024 Mar 13;19(3):e0299401. doi: 10.1371/journal.pone.0299401 (PMC10936848; doi:10.1371/journal.pone.0299401)

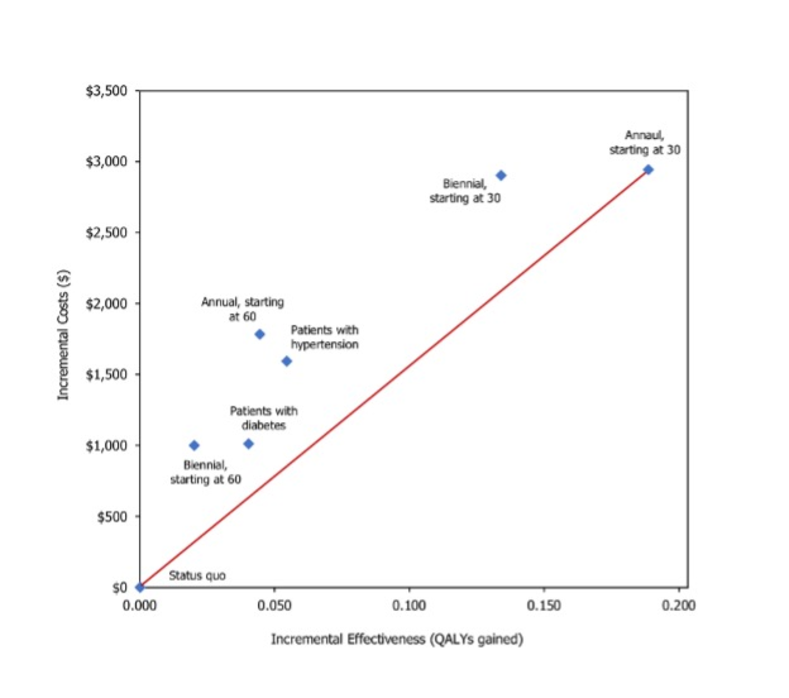

Supplement: S1 Fig — Annual universal screening starting at the age of 30 is the non-dominated screening policy, followed closely by screening the patients with diabetes. (TIF) [file pone.0299401.s001.tif]

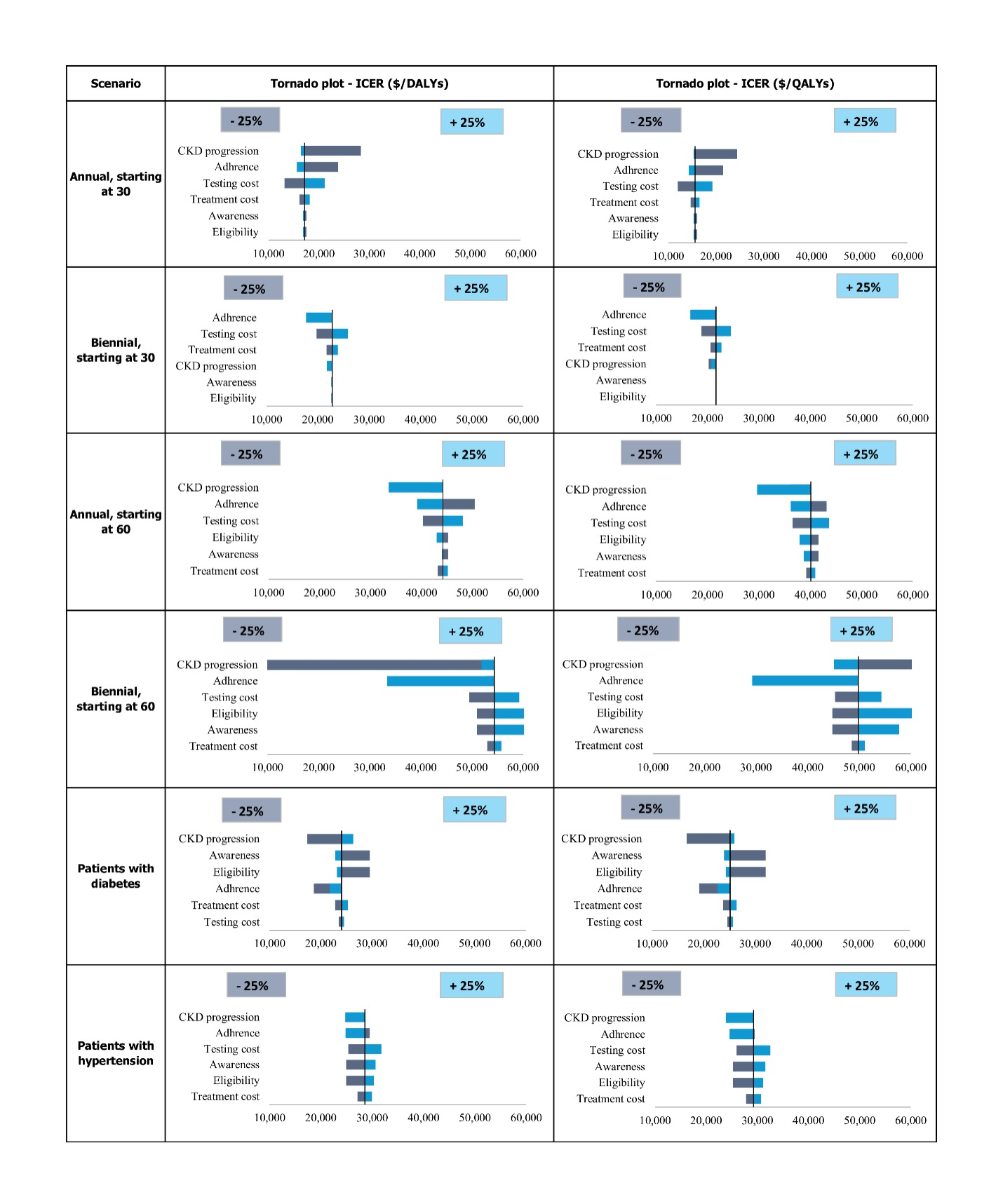

Supplement: S2 Fig — Parallel analysis using DALYs and QALYs resulted in analogous results. (TIF) [file pone.0299401.s002.tif]

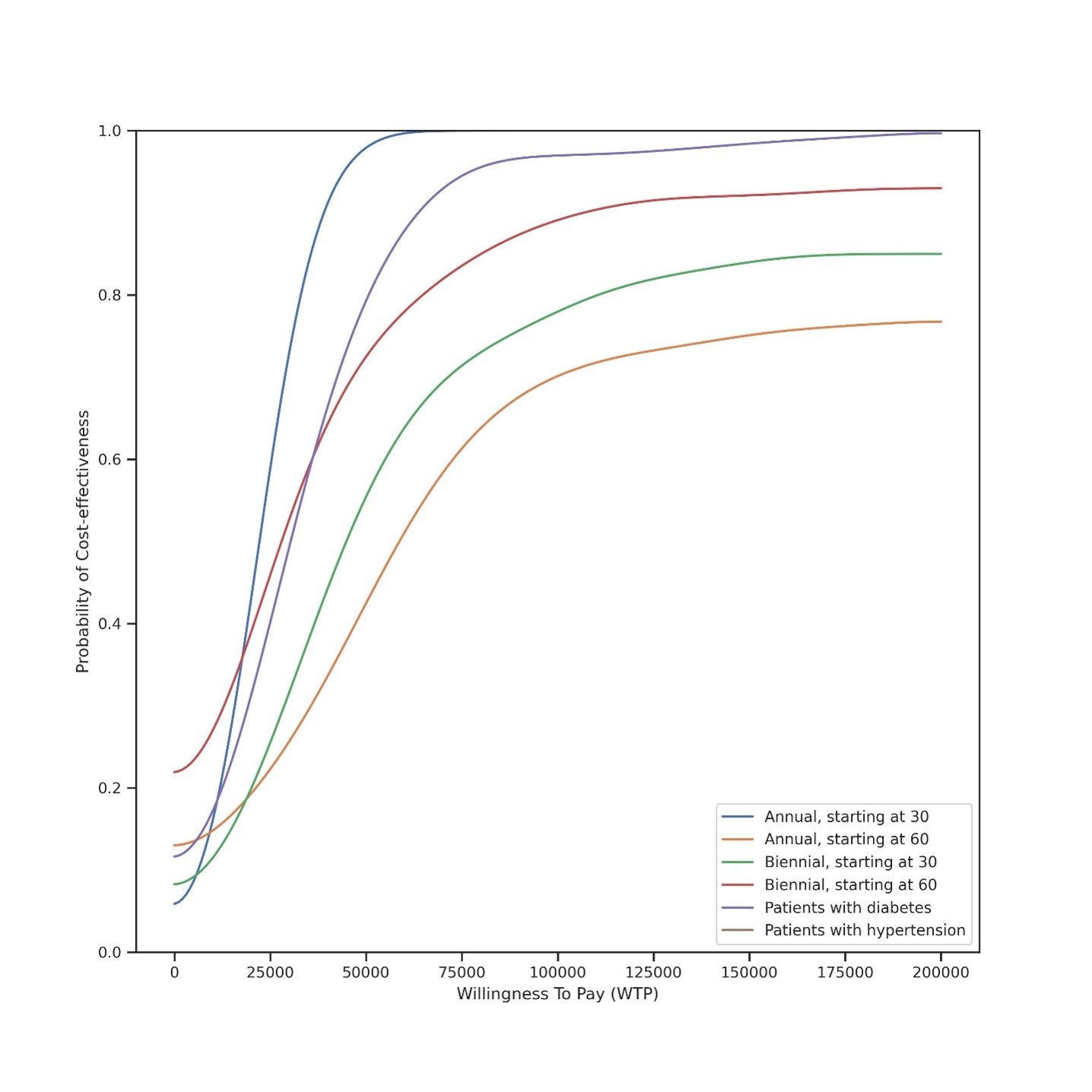

Supplement: S3 Fig — Universal annual screening for individuals 30 and older has the highest probability of being cost-effective for the willingness-to-pay values of $24,000/DALY-averted and above. For enhanced clarity, the curves have been subjected to smoothing using a Gaussian filter. (TIF) [file pone.0299401.s003.tif]
