# Supplementary material for: Cost-effectiveness of screening for chronic kidney disease using a cumulative eGFR-based statistic
Source: PLoS One. 2024 Mar 13;19(3):e0299401. doi: 10.1371/journal.pone.0299401 (PMC10936848; doi:10.1371/journal.pone.0299401)
Supplement: S1 Appendix — (DOCX) [file pone.0299401.s004.docx]

**SUPPLEMENTARY MATERIALS**

**Supplement S1. Detailed Model Parameters for the CKD Screening Model**

**Table S1. Natural history model parameters.**

| **Model Parameter** | **Parameter Value** | **Source** |
| --- | --- | --- |
| Micro-simulation and cohort |  |  |
| Initial age | 30 | [17] |
| Race/ethnicity distribution |  |  |
| White | 0.357 (♀); 0.365 (♂) |  |
| Black | 0.067 (♀); 0.060 (♂) |  |
| Hispanic | 0.072 (♀); 0.078 (♂) |  |
| Natural history model |  |  |
| Initial eGFR at age 30 | 101.9 ± 19.3 | [17] |
| Risk factors and complications |  |  |
| Proteinuria |  | [17] |
| Prevalence at age 30 in individuals with: |  |  |
| DM | 0.020 (♀);0.020 (♂) |  |
| HTN | 0.035 (♀);0.014 (♂) |  |
| Neither | 0.014 (♀); 0.005 (♂) |  |
| Incidence rate in individuals with: |  |  |
| DM | 0.048 - 0.048* |  |
| HTN | 0.017 - 0.026 |  |
| Neither | 0.002 - 0.053 |  |
| DM |  | [17,21] |
| Prevalence at age 30 in individuals who are: |  |  |
| White | 0.008 (♀); 0.016 (♂) |  |
| Black | 0.047 (♀); 0.007 (♂) |  |
| Hispanic | 0.026 (♀);0.002 (♂) |  |
| Incidence rate in individuals who are*: |  |  |
| White | (0.003 - 0.012) |  |
| Black | (0.004 - 0.016) |  |
| Hispanic | (0.003 - 0.014) |  |
| HTN (defined as SBP ≥ 140 mmHg) |  | [17,21] |
| average SBP in individuals with |  |  |
| CKD | (100 - 192) (♀)  (102 - 183) (♂) |  |
| No CKD | (96 - 189) (♀)  (104 - 181) (♂) |  |
| Anemia** |  | [20,22,23] |
| Prevalence at age 30 in individuals with: |  |  |
| CKD Stage 3A, and | 0.053 (DM); 0.038 (No DM) |  |
| CKD Stage 3B, and | 0.053 (DM); 0.038 (No DM) |  |
| CKD Stage 4, and | 0.024 (DM); 0.019 (No DM) |  |
| CKD Stage 5, and | 0.024 (DM); 0.019 (No DM) |  |
| Incidence rate in individuals with*: |  |  |
| CKD Stage 3A, and | (0.014 – 0.16) (DM)  (0.017 – 0.09) (No DM) |  |
| CKD Stage 3B, and | (0.014 – 0.16) (DM)  (0.017 – 0.09) (No DM) |  |
| CKD Stage 4, and | (0.083 – 0.57) (DM)  (0.098 – 0.41) (No DM) |  |
| CKD Stage 5, and | (0.083 – 0.57) (DM)  (0.098 – 0.41) (No DM) |  |
| Mortality |  | [24,25] |
| Annual all-cause mortality (including CVD mortality) | Varies by age, sex, race/ethnicity |  |
| ESRD mortality | Varies by age, sex, race/ethnicity, DM, HTN |  |
| Life expectancy | Varies by age, sex, race/ethnicity |  |
| Annual expected eGFR decrease (mL/min/1.73 m^2^) for individuals with: |  | [17] |
| DM, and |  |  |
| Proteinuria | 0.65 (eGFR ≥ 60)  0.65 (eGFR < 60) |  |
| No proteinuria | 0.72 (eGFR ≥ 60)  4.2 (eGFR < 60) |  |
| HTN, and |  |  |
| Proteinuria | 0.72 (eGFR ≥ 60)  1.4 (eGFR < 60) |  |
| No proteinuria | 0.78 (eGFR ≥ 60)  3.9 (eGFR < 60) |  |
| Neither, but with |  |  |
| Proteinuria | 1.1 (eGFR ≥ 60)  2.8 (eGFR < 60) |  |
| No proteinuria | 4.1 (eGFR ≥ 60)  5.2 (eGFR < 60) |  |

* Ranges shown reflect variation by age, sex and race/ethnicity.

** CKD-caused Anemia only occurs in CKD stages 3 to 5.

Abbreviations: ♀, female; ♂, male; DM, diabetes mellitus; eGFR, estimated glomerular filtration rate; ESRD, end-stage renal disease; HTN, hypertension.

**Table S2. Disability weights CKD stage and presence of anemia^*^**

|  | **No Anemia** | **Mild Anemia** | **Moderate Anemia** | **Severe Anemia** |
| --- | --- | --- | --- | --- |
| **No CKD** | 0 | 0 | 0 | 0 |
| **CKD stage 1** | 0 | 0 | 0 | 0 |
| **CKD stage 2** | 0 | 0 | 0 | 0 |
| **CKD stage 3A** | 0 | 0.004 | 0.052 | 0.149 |
| **CKD stage 3B** | 0 | 0.004 | 0.052 | 0.149 |
| **CKD stage 4** | 0.104 | 0.108 | 0.150 | 0.237 |
| **CKD stage 5** | 0.569 | 0.570 | 0.591 | 0.631 |
| **ESKD, first year** | 0.571 | 0.571 | 0.571 | 0.571 |
| **ESKD, second year and on** | 0.412 | 0.412 | 0.412 | 0.412 |
| **Deceased** | 1 | 1 | 1 | 1 |

**^*^** Disability weights were estimated from published study [32].

**Table S3. Coefficients of regression model for cost estimates.**

| **Model Parameter** | **Parameter Value** | **Source** |
| --- | --- | --- |
| No CKD (interception) | $1,033 | [18,29,30] |
| CKD stage 1 | $5,200 |  |
| CKD stage 2 | $6,466 |  |
| CKD stage 3A | $6,673 |  |
| CKD stage 3B | $6,673 |  |
| CKD stage 4 | $8,250 |  |
| CKD stage 5 | $36,491 |  |
| ESKD, first year | $89,868 |  |
| ESKD, second year and on | $74,484 |  |
| DM | $2,147 |  |
| DM × CKD stage 1 | $(554) |  |
| DM × CKD stage 2 | $(554) |  |
| DM × CKD stage 3A | $402 |  |
| DM × CKD stage 3B | $402 |  |
| DM × CKD stage 4 | $4,710 |  |
| DM × CKD stage 5 | $20,835 |  |
| HTN | $1,358 |  |
| HTN × CKD stage 1 | $(1,604) |  |
| HTN × CKD stage 2 | $(1,604) |  |
| HTN × CKD stage 3A | $(2,390) |  |
| HTN × CKD stage 3B | $(2,390) |  |
| HTN × CKD stage 4 | $(3,581) |  |
| HTN × CKD stage 5 | $(15,841) |  |
| Anemia | $8,391 |  |
| Anemia × CKD stage 1 | $1,407 |  |
| Anemia × CKD stage 2 | $1,407 |  |
| Anemia × CKD stage 3A | $(2,310) |  |
| Anemia × CKD stage 3B | $(2,310) |  |
| Anemia × CKD stage 4 | $(5,689) |  |
| Anemia × CKD stage 5 | $(25,166) |  |
| Proteinuria | $5,672 |  |
| Age | $42 |  |
| Male | $(243) |  |

**Supplement S2. Additional Cost-effectiveness Analysis Results**

**Incremental cost-effectiveness analysis**

The incremental cost-effectiveness analysis is provided in **Table S4**. Analysis based on DALYs and QALYs as effectiveness outcomes respectively showed similar results, leading to identical finding that the universal annual screening starting at the age of 30 was the non-dominated policy on the cost-effectiveness frontier.

**Table S4: Results of incremental cost-effectiveness analysis**

| **Screening policy** | **DALYS** | **QALYS** | **Costs** | **ICER ($/DALY)** | **ICER ($/QALY)** | **Incremental ICER ($/DALY)** | **Incremental ICER ($/QALY)** |
| --- | --- | --- | --- | --- | --- | --- | --- |
|  |  |  |  |  |  |  |  |
| **Status quo** | 3.576 | 23.605 | $123,132.71 | – | – | – | – |
| **Biannual, starting at 60** | 3.557 | 23.625 | $124,133.46 | $54,372.97 | $49,792.88 | Dominated | Dominated |
| **Patients with diabetes** | 3.534 | 23.645 | $124,144.46 | $24,053.62 | $25,148.36 | Dominated | Dominated |
| **Annual, starting at 60** | 3.535 | 23.650 | $124,916.01 | $44,242.04 | $40,123.97 | Dominated | Dominated |
| **Patients with hypertension** | 3.520 | 23.660 | $124,725.93 | $28,765.97 | $27,670.97 | Dominated | Dominated |
| **Biannual, starting at 30** | 3.448 | 23.739 | $126,036.13 | $22,816.93 | $21,680.06 | Dominated | Dominated |
| **Annual, starting at 30** | 3.404 | 23.794 | $126,076.75 | $17,163.17 | $15,614.33 | $17,163.17 | $15,614.33 |

**One-way sensitivity analysis**

The results of one-way sensitivity analyses are provided in **Figures S2**. Since incremental cost-effectiveness ratio (ICER) is sensitive to the magnitude of incremental costs and incremental effects, the impact of one-way sensitivity analysis on health and cost outcomes are also provided in **Figure S3**.

**Probabilistic Sensitivity Analysis**

The probability distribution of parameters used in the probabilistic sensitivity analysis are listed in **Table S5**. **Figure S3** shows the probability that each CUSUM_GFR_-based screening policy is cost-effective under various of willingness-to-pay values when compared with the same comparator of the status quo. Universal annual screening for individuals 30 and older has the highest probability of being cost-effective for the willingness-to-pay values of $24,000/DALY-averted and above.

**Table S5: Sensitivity analysis parameters**

| **Variable** | **Value** | **Distribution** | **Parameters** | **Source** |
| --- | --- | --- | --- | --- |
| CKD progression rate | Table 1 | Triangular | (0.75, 1, 1.25) × value | (17,36,37) |
| Awareness | Table 1 | Triangular | (0.75, 1, 1.25) × value | (14,36,37) |
| Eligibility | Table 1 | Triangular | (0.75, 1, 1.25) × value | (36–38) |
| Adherence | Table 1 | Triangular | (0.75, 1, 1.25) × value | (16,36) |
| Testing cost | Table 1 | Gamma | Mean = value,  SD = 25% × value | (36) |
| Treatment cost | Table 1 | Gamma | Mean = value,  SD = 25% × value | (36) |
